# PCSK9 acts as a key regulator of Aβ clearance across the blood–brain barrier

**DOI:** 10.1007/s00018-022-04237-x

**Published:** 2022-03-27

**Authors:** Alexander D. Mazura, Anke Ohler, Steffen E. Storck, Magdalena Kurtyka, Franka Scharfenberg, Sascha Weggen, Christoph Becker-Pauly, Claus U. Pietrzik

**Affiliations:** 1 Institute of Pathobiochemistry, University Medical Center of the Johannes Gutenberg-University Mainz, Duesbergweg 6, 55128 Mainz, Germany; 2 Institute of Biochemistry, Christian Albrecht University of Kiel, 24098 Kiel, Germany; 3 Department of Neuropathology, Heinrich Heine University Düsseldorf, 40225 Düsseldorf, Germany

**Keywords:** Alzheimer’s disease, Amyloid-beta, Blood–brain barrier, Low-density lipoprotein receptor-related protein 1, Proprotein convertase subtilisin/kexin type 9, Monoclonal antibody therapy

## Abstract

Despite the neurodegenerative disorder Alzheimer’s disease (AD) is the most common form of dementia in late adult life, there is currently no therapy available to prevent the onset or slow down the progression of AD. The progressive cognitive decline in AD correlates with a successive accumulation of cerebral amyloid-β (Aβ) due to impaired clearance mechanisms. A significant percentage is removed by low-density lipoprotein receptor-related protein 1 (LRP1)-mediated transport across the blood–brain barrier (BBB) into the periphery. Circulating proprotein convertase subtilisin/kexin type 9 (PCSK9) binds to members of the low-density lipoprotein receptor protein family at the cell surface and targets them for lysosomal degradation, which reduces the number of functional receptors. However, the adverse impact of PCSK9 on LRP1-mediated brain Aβ clearance remains elusive. By using an established BBB model, we identified reduced LRP1-mediated brain-to-blood Aβ clearance due to PCSK9 across different endothelial monolayer in vitro. Consequently, the repetitive application of FDA-approved monoclonal anti-PCSK9 antibodies into 5xFAD mice decreased the cerebral Aβ burden across variants and aggregation state, which was not reproducible in brain endothelial-specific *LRP1*^*−/−*^ 5xFAD mice. The peripheral PCSK9 inhibition reduced Aβ pathology in prefrontal cortex and hippocampus–brain areas critically involved in memory processing—and prevented disease-related impairment in hippocampus-dependent memory formation. Our data suggest that peripheral inhibition of PCSK9 by already available therapeutic antibodies may be a novel and easily applicable potential AD treatment.

**Supplementary Information:**

The online version contains supplementary material available at 10.1007/s00018-022-04237-x.

## Introduction

Sporadic Alzheimer’s disease (AD) is a progressive neurodegenerative disorder and the most common form of dementia in late adult life, but there is still no treatment to prevent or slow down the progression of AD available [1].

The progressive loss of cognitive functions is typically associated with the accumulation of extracellular amyloid-β (Aβ) peptides in the brain [2]. According to the Aβ cascade hypothesis, the development of AD is due to an imbalance between the production and clearance of cerebral Aβ [3]. Increased concentrations of soluble Aβ oligomers are associated with disrupted synaptic plasticity and neuronal degeneration affecting memory formation and function [4–8].

Under physiological conditions brain Aβ clearance exceeds its production [9], but has been shown to be decreased in AD patients without changes in production rate [10]. Many lines of evidence suggest that impaired brain clearance of Aβ drives the onset and progression of late-onset AD [3]. An important clearance pathway for cerebral Aβ is the steady transport across the blood–brain barrier (BBB) [11]. To overcome the highly selective BBB most molecules require carrier- or receptor-mediated transport systems [12]. Directed transport is achieved by the polarity of the endothelial cells with differential receptor composition of brain- (abluminal) and blood-faced (luminal) membrane [12]. The brain-to-blood transfer of monomeric Aβ is highly dependent on low-density lipoprotein receptor-related protein 1 (LRP1) [13, 14], which in brain endothelium is primarily located at the abluminal side [13, 15]. LRP1 is expressed in a variety of cell types in the brain and the periphery including abundant expression levels in the liver [16], largely responsible for the rapid endocytosis and subsequent elimination of peripheral Aβ [17–19].

LRP1 belongs to the family of low-density lipoprotein (LDL) receptors, which is post-transcriptionally regulated by proprotein convertase subtilisin/kexin type 9 (PCSK9), a member of the proteinase K subfamily of subtilases [20–22]. PCSK9 is highly expressed in the liver as well as in kidney, small intestine and the brain and circulates in the blood [23]. The mode of extracellular PCSK9 regulation is well studied for the eponym of the LDL receptor protein family and its ligand LDL-cholesterol. Briefly, cell surface LDL receptor regulates the cholesterol concentration in the serum by constant binding and internalization of LDL-cholesterol (LDL-C) particles [24]. Subsequently, the LDL receptor/LDL-C complex dissociates in the endosomal-lysosomal system [25], allowing the receptor to recycle back to the cell surface [24]. Extracellular PCSK9 is able to bind to the membrane-anchored LDL receptor and enters in complex with the LDL receptor the endosomal-lysosomal system [26]. But instead of complex dissociation, the binding between PCSK9 and the LDL receptor is strengthened with decreasing pH resulting in the lysosomal degradation of the LDL receptor rather than entering the recycling pathway [26]. Due to this correlation, peripheral PCSK9 inhibition with monoclonal antibodies is an established therapy to reduce the plasma cholesterol level in patients suffering from hypercholesterolemia by increasing the LDL receptor density at the cell surface [27].

Although the mode of PCSK9-mediated LDL receptor downregulation has been extensively studied, its role on other LDLR family members is less well understood. In line with several cohort studies including the Rotterdam Study [28], which have shown that cholesterol-lowering medications in midlife were associated with a 50–70% lower risk of late-life development of AD [29], we hypothesized that the inhibition of extracellular PCSK9 by FDA-approved monoclonal antibodies might strengthen LRP1-mediated Aβ clearance and thus be a potential therapeutic approach to AD.

## Methods

### Study design

In this study, we used our established in vitro BBB model to assess the influence of PCSK9 on LRP1-mediated brain-to-blood Aβ transcytosis across different endothelial monolayer [14, 30]. Our system allows the usage of Aβ_1–42_ in physiological concentrations to avoid potential Aβ toxicity [31]. Initially, the immortalized mouse brain capillary endothelial cell line bEnd.3 was used due to its well-studied receptor-mediated transport system and expression of tight junction proteins producing a decent paracellular tightness [32–34]. By generating PCSK9-producing bEnd.3 cells the overall PCSK9 influence on Aβ clearance was investigated. The application of LRP1-blocking antibody 11E2 was used to determine the significance of cell surface LRP1 within this regulatory mechanism [35]. Subsequently, we used brain mouse endothelial cells (BMECs) harboring an inducible LRP1 knock-out and recombinant PCSK9 to analyze the regulatory impact of extracellular PCSK9 on LRP1-mediated Aβ clearance across primary endothelial cells [14]. Porcine brain endothelial cells (PBECs) form even higher in vitro transendothelial electric resistance values in comparison to BMECs a central characteristic of a functional BBB and were used to verify for PCSK9-regulated BBB transport across species [36, 37]. Based on our in vitro observations, we investigated the influence of systemic PCSK9 inhibition via monoclonal antibodies on several AD characteristics using the mouse model 5 × familial Alzheimer’s disease (FAD). Female 5xFAD mice display a considerable Aβ_1–42_ burden, distinct plaque pathology and impaired cognitive capacities already at 6 months of age [38, 39], which were detected via high-resolution urea SDS-PAGE [40], ELISA, immunofluorescent plaque load analyses, and a slightly adapted established fear conditioning paradigm [39, 41]. By inducing a brain endothelial-specific LRP1 knock-out in 5xFAD mice, we tested our hypothesis regarding PCSK9 downregulation of brain endothelial LRP1 function in vivo [14]*.* The administration of FDA-approved monoclonal anti-PCSK9 antibody Alirocumab was based on human therapy strategy and designed less severe regarding concentration and treatment period in relation to comparable animal studies to ensure tolerability [42, 43]. As a consequence of the patent dispute between Amgen and Sanofi concerning the PCSK9 inhibitors Evolocumab and Alirocumab, we had to switch for a subset of experiments from the previously used PCSK9 inhibitor Alirocumab to Evolocumab, which is specified in the respective figure legends. Before the start of any treatment, seeded endothelial cells with similar capacity values and mice with similar body weight were randomized into groups in all experiments. The number of replicates and independent experimental runs as well as blinded evaluation are indicated in the respective figure legends.

### Animals, treatment, and isolation of animal material

If not stated otherwise, mice were housed on a 12-h-light cycle and had ad libitum access to water and standard laboratory diet. To study PCSK9 inhibition on cerebral Aβ burden and learning behavior, heterozygous 5xFAD mice (Tg6799; > 10 backcrosses with C57BL/6J mice; kindly provided from Thomas A. Bayer; Georg-August-University Göttingen, Germany) as described before [38] were bred to *Slco1c1-CreER*^*T2 TG/wt*^ and *LRP1*^*fl/fl*^ mice, which introduced a tamoxifen-inducible LRP1 brain endothelial-specific knockout (*LRP1*^*BE−/−*^) into the AD model [14].

14-weeks-old female 5xFAD transgenic or wildtype littermates were randomized into groups with similar average body weights (18–20 g), respectively. 1 µg/g Alirocumab (Sanofi), Evolocumab (Amgen), or non-specific human IgG2 control antibody (Bio X Cell) in 0.9% isotonic saline solution (BBraun) or the analogous volume of pure 0.9% isotonic saline solution were weekly i.p. injected for a period of 10 weeks. 7 days before starting the injection procedure *LRP1*^*BE−/−*^ was induced by i.p. injection of 75 µg/g tamoxifen (Sigma-Aldrich) for 5 consecutive days and maintained with chow containing 400 mg/kg tamoxifen citrate (LASvendi). To sacrifice animals, mice were anesthetized with 100 µg/g ketamine (Pfizer) in combination with 10 µg/g xylazine (Bayer HealthCare) and euthanized by cervical dislocation.

BMECs from 12- to 15-week-old wildtype 5xFAD littermates with *Slco1c1-CreER*^*T2 wt/wt*^ or ^*TG/wt*^ and *LRP1*^*fl/fl*^ were isolated and cultured following an established protocol [36]. Briefly, meninges of isolated forebrains were removed and tissue subsequently mechanically dissociated. Digestion was conducted using 100 U/ml collagenase CLS2 (Worthington) and 10 U/ml DNase (Sigma-Aldrich) in DMEM, high glucose (Gibco) for 1 h (200 rpm, 37 °C). Subsequently, the homogenate was centrifuged for 8 min (1000 rcf, 4 °C). Myelin was removed by resuspending the pellet in 20% (w/v) BSA (Roth) in DMEM, high glucose followed by centrifugation for 20 min (1000 rcf, 4 °C). The pellet was further digested in DMEM, high glucose containing 0.1 U/ml collagenase in combination with 0.8 U/ml dispase (Roche) and 10 U/ml DNase for 1 h (200 rpm, 37 °C). Microvascular endothelial capillaries were isolated using a 33% continuous Percoll (GE Healthcare) gradient centrifuged for 10 min (1000 rcf, 4 °C) without brakes and directly plated on 24-well Transwell filters (pore size, 0.4 μm; surface area, 33.6 mm^2^; Greiner Bio-One) coated with a mixture of 400 µg/ml collagen IV and 100 µg/ml fibronectin (both from Sigma-Aldrich). Endothelial cells were cultured in DMEM, high glucose supplemented with 20% (v/v) plasma-derived bovine serum (First Link), 100 U/ml penicillin/100 μg/ml streptomycin, 2 mmol/l l-glutamine (both from Gibco), 4 μg/ml puromycin (Alexis), and 30 μg/ml endothelial cell growth supplement (Sigma-Aldrich) at 37 °C and 5% CO_2_. After 2 days in culture puromycin was withdrawn. The brain endothelial-specific LRP1 knockout was induced by applying 1 µmol/l 4-Hydroxytamoxifen (Sigma-Aldrich) to the culture media for 2 consecutive days.

Pig brains were purchased from a local slaughterhouse and stored in ice-cold artificial cerebrospinal fluid (CSF) [124 mmol/l NaCl, 26 mmol/l NaHCO_3_, 3 mmol/l KCl, 2 mmol/l CaCl_2_, 2 mmol/l MgSO_4_, 1.25 mmol/l NaH_2_PO_4_, and 10 mmol/l glucose equilibrated with carbogen to pH 7.4] until further processing. PBECs were isolated and cultured as described previously [44]. Briefly, meninges, large surface vessels, and choroid plexus were removed thoroughly. Brains were mechanically dissociated and digested using 300 U/ml dispase (Corning) in Medium 199, containing 0.7 mmol/l l-glutamine, 500 μg/ml gentamycin (all from Sigma-Aldrich), and 100 U/ml penicillin/100 μg/ml streptomycin for 2 h (200 rpm, 37 °C). After centrifugation for 10 min (1000 rcf, 4 °C) the pellet was resuspended thoroughly in Medium 199, supplemented with 18% dextran (~ 160,000 g/mol; Roth) and centrifuged for 10 min (6800 rcf, 4 °C) to remove myelin. The pellet was resuspended in Medium 199, containing 0.7 mmol/l l-glutamine, 500 μg/ml gentamycin, and 100 U/ml penicillin/100 μg/ml streptomycin and filtered (180 µm nylon mesh) before further digestion with 0.1 U/ml collagenase in combination with 0.8 U/ml dispase for 1 h (200 rpm, 37 °C). The homogenate was centrifuged for 10 min (110 rcf) and the pellet resuspended in Medium 199, containing 0.7 mmol/l l-glutamine, 500 μg/ml gentamycin, 100 U/ml penicillin﻿/100 μg/ml streptomycin, and 10% (v/v) horse serum (Sigma-Aldrich). Microvascular endothelial capillaries were isolated using a discontinuous Percoll (Amersham Pharmacia Biotech) gradient consisting of 44.44% (v/v) 1.03 g/ml and 33.33% (v/v) 1.07 g/ml Percoll, centrifuged for 10 min (1300 rcf, 4 °C) without brakes and plated on flasks coated with 133.33 µg/ml collagen G (Sigma-Aldrich). Endothelial cells were cultured in DMEM, high glucose supplemented with 20% (v/v) plasma-derived bovine serum (First Link), 100 U/ml penicillin/100 µg/ml streptomycin, 0.7 mmol/l l-glutamine, and 4 µg/ml puromycin at 37 °C and 5% CO_2_. After 2 days in culture puromycin was withdrawn. Endothelial cells were frozen in culture media but containing 20% (v/v) horse serum and 10% (v/v) DMSO and stored in liquid nitrogen until further use (passaged only once) or plated directly on 24-well Transwell filters (pore size, 0.4 μm; surface area, 33.6 mm^2^) coated with a mixture of 400 µg/ml collagen IV and 100 µg/ml fibronectin.

### Cell culture and transfections

The mouse brain endothelial cell line bEnd.3 (ATCC) and GP2-293 cells (Clontech) were cultured in DMEM, high glucose supplemented with 10% (v/v) fetal bovine serum (Gibco) and 100 U/ml penicillin/100 µg/ml streptomycin at 37 °C and 5% CO_2_. The culture media of GP2-293 was further supplemented with 1 mmol/l sodium pyruvate (Sigma-Aldrich).

To generate bEnd.3 cells stably expressing PCSK9, the packaging cell line GP2-293 was transiently transfected with pLBCX-PCSK9 (cDNA kindly provided by Jay Horton; UT Southwestern, USA) and pVSV-G (ratio 1:1; AddGene) via polyethylenimine (Polysciences) to initiate viral production. DNA and polyethylenimine (ratio 1:4) were diluted in 30 µl/µg Opti-MEM (Gibco), respectively, mixed and added after 15 min to the cells for 4 h. Long-term cell adherence of GP2-293 was ensured by poly-l-ornithine (Sigma-Aldrich) coating. Virus-enriched media was collected 24 h after media change and subsequent infection with viral particles occurred for 24 h in the presence of 50 µg/ml polybrene (Millipore). bEnd.3 cells were selected using 8 µg/ml blasticidin (Invivogen) and stable pools were verified for adequate PCSK9 expression by immunoblotting.

### Antibodies

Alirocumab (Sanofi), Evolocumab (Amgen), or non-specific human IgG2 control antibody (BP0301; Bio X Cell) was diluted in 0.9% isotonic saline solution and i.p. applied at a final concentration of 1 µg/g bodyweight. In vitro, blocking of LRP1 function was achieved by using 15 µg/ml mouse anti-11E2 antibody [35] in comparison to equal amounts of a non-specific mouse IgG control (12-371; Sigma-Aldrich). Human Aβ was immunoprecipitated and detected by mouse anti-IC16 [45] and the α-chain/full length of LRP1 recognized by mouse anti-11E2 antibody [14]. Rabbit anti-1704 antibody was used to identify the LRP1 β-chain [46]. Rabbit anti-LDL receptor antibody 3/43 was kindly provided by Joachim Herz (UT Southwestern, USA). Rabbit anti-PCSK9 (ab125251), rabbit anti-Apolipoprotein E (ApoE) (ab150032), chicken anti-rabbit (ab6831), chicken anti-mouse (ab6706), HRP-conjugated donkey anti-chicken (ab16349), and Alexa488-conjugated goat anti-mouse antibody (ab150117) were purchased from Abcam. Rabbit anti-β-actin (A2066) and HRP-conjugated goat anti-rabbit antibody (A5278) were purchased from Sigma-Aldrich. HRP-conjugated donkey anti-mouse antibody (715–035-151) was purchased from Dianova.

### Protein extraction, SDS-PAGE, immunoblotting, and ELISA

Extracted blood was supplemented with an EDTA-free protease inhibitor cocktail (Roche Applied Science) and allowed to clot at room temperature for 10 min. Liver were weighed and homogenized in 3 ml/g fresh cold tissue lysis buffer [50 mmol/l Tris (pH 7.4), 150 mmol/l NaCl, 0.1% (w/v) SDS, 1% (v/v) Nonidet P-40, 0.5% (w/v) sodium deoxycholate, 1 mmol/l EDTA (pH 7.4)] containing an EDTA-free protease inhibitor cocktail using the Precellys Lysing Kit (Bertin instruments). Extracts were purified for 3 min (18,620 rcf, 4 °C). CSF samples were isolated via puncture of cisterna magna as described previously [47] and purified for 10 min (1000 rcf, 4 °C). Brains were weighed and soluble and insoluble brain fractions sequential extracted. Initially, homogenization by a glass homogenizer occurred in 6 ml/g PBS, containing an EDTA-free protease inhibitor cocktail and 1 mmol/l EDTA (pH 7.4). The homogenate was centrifuged for 20 min (131,700 rcf, 4 °C) and the supernatant (soluble brain fraction) isolated. Subsequently, the brain pellet was homogenized in 4 ml/g PBS containing 2% (v/v) SDS using the Precellys Lysing Kit. Centrifugation was repeated and the supernatant (insoluble brain fraction) collected. The preparation of capillary-depleted brain fractions based on an established protocol [48]. Briefly, brains were weighed, homogenized by a glass homogenizer in 6 ml/g PBS, and centrifuged for 5 min (1350 rcf, 4 °C). The pellet was resuspended in 3 ml/g PBS, containing 18% dextran (~ 70,000 g/mol; Roth), vortexed thoroughly for 1 min, and centrifuged for 10 min (4863 rcf, 4 °C). Subsequently, the supernatant (capillary-free brain fraction) was transferred, mixed thoroughly with 3 ml/g PBS, and centrifuged for 5 min (1350 rcf, 4 °C). The brain parenchymal pellet was lysed for 20 min using tissue lysis buffer containing an EDTA-free protease inhibitor cocktail and 1 mmol/l EDTA (pH 7.4) and purified for 20 min (18,620 rcf, 4 °C). Each isolated sample was snap frozen via liquid nitrogen and stored at − 80 °C.

Protein concentrations were determined using the Pierce™ BCA Protein Assay Kit (Thermo Fisher Scientific) according to the manufacturer’s protocol. Protein samples were supplemented with Roti^®^-Load 1 SDS sample buffer (Roth), boiled for 5 min at 95 °C, and subjected to SDS-PAGE using 8% T/3.3% C Tris–Glycine gels (BioRad). Subsequently, proteins were transferred onto nitrocellulose membranes (GE Healthcare), which were blocked with TBS containing 0.1% (v/v) Tween 2﻿0 and 5% (w/v) non-fat dry milk for 1 h.

Precleared serum via raw Protein A Agarose (Roche) was diluted in PBS containing an EDTA-free protease inhibitor cocktail and incubated with Protein A Agarose crosslinked to Evolocumab overnight at 4 °C. Beads were washed three times with PBS, followed by elution in 100 mmol/l glycine (pH 2.7) and neutralization with 500 mmol/l tris (pH 8.0).

Immunoprecipitation of cerebral Aβ from soluble and insoluble brain fractions were performed following a protocol described before [49]. Briefly, 1 mg of soluble or 500 µg of insoluble brain fraction was added to IP detergent buffer [50 mmol/l HEPES (pH 7.4), 150 mmol/l NaCl, 0.05% (w/v) SDS, 0.5% (v/v) Nonidet P-40, 1 mmol/l EDTA (pH 7.4)], containing an EDTA-free protease inhibitor cocktail and incubated with Dynabeads™ M-280 sheep anti-mouse IgG (Invitrogen) precoated with IC16 antibody overnight at 4 °C. Beads were washed twice with PBS containing 1% BSA and boiled at 95 °C for 5 min in 15 µl urea SDS-PAGE loading buffer [0.36 mol/l bis–Tris, 0.16 mol/l bicine, 1% (w/v) SDS, 15% (w/v) sucrose and 0.0075% (w/v) bromophenol blue] to release Aβ peptides. Separation of immunoprecipitated Aβ_1–40_ and Aβ_1–42_ was performed by high-resolution urea SDS-PAGE using 8 mol/l urea (AppliChem) 10% T/5% C Bicine-Tris gels (BioRad) following an established protocol [40]. Different Aβ species were verified by loaded peptide standards purchased from Genosphere. Proteins were transferred onto PVDF membranes (Immobilion-P), which were subsequently boiled for 3 min in PBS and blocked with TBS containing 0.1% (v/v) Tween 20 and 5% (w/v) non-fat dry milk for 30 min.

Incubation with primary and secondary antibodies was conducted overnight or for 1 h, respectively, and chemiluminescence was generated using Immobilon Western HRP Substrate (Millipore).

The levels of Aβ_1–40_ and Aβ_1–42_ in soluble and insoluble brain fractions were blindly determined using Human Amyloid β (FL) Assay Kits (Immuno-Biological Laboratories) according to the manufacturer’s protocol.

Serum VLDL/LDL and HDL cholesterol were separated and concentrations blindly determined using the Cholesterol Assay Kit—HDL and LDL/VLDL (Abcam) according to the manufacturer’s protocol.

### In vitro transcytosis studies of [^125^I]-Aβ_1–42_

Cells or microvascular endothelial capillaries subjected for in vitro Aβ transcytosis studies were plated on 24-well Transwell filters (pore size, 0.4 μm; surface area, 33.6 mm^2^) coated with a mixture of 400 µg/ml collagen IV and 100 µg/ml fibronectin and cultivated in a cellZscope device (nanoAnalytics) at 37 °C and 5% CO_2_. When each cell layer generates a capacitance < 10 nF/mm^2^, high transendothelial electric resistance values (bEnd.3: ≥ 0.5 Ω/mm^2^, BMECs: ≥ 0.8 Ω/mm^2^, PBECs: ≥ 3 Ω/mm^2^) were induced by replacing the culture media for 24 h (bEnd.3 cells, BMECs) or 48 h (PBECs) with DMEM/Ham's F-12 (Gibco), containing 100 U/ml penicillin/100 μg/ml streptomycin, 1 mmol/l l-glutamine (bEnd.3 cells, BMECs) or 0.7 mmol/l l-glutamine (PBECs), and 550 nmol/l hydrocortisone (Sigma-Aldrich) as described previously [36]. Subsequently, media was exchanged by fresh media with or without 5 µg/ml recombinant PCSK9, 15 µg/ml LRP1-blocking antibody 11E2, or equal amounts of non-specific mouse antibody and replaced after 2 h. To study brain (abluminal)-to-blood (luminal) transcytosis, the abluminal compartment was further supplemented with 0.1 nmol/l [^125^I]-Aβ_1–2_ and 37 kBq/ml [^14^C]-inulin (both from PerkinElmer) as a marker for paracellular diffusion. After 45 min of incubation, the level of transported [^125^I]-Aβ_1–42_ and diffused [^14^C]-inulin within the luminal compartment were determined. Media was supplemented with 7.5% (v/v) TCA, followed by incubation and centrifugation for 10 min (20,817 rcf, 4 °C). TCA-pellets, containing intact [^125^I]-Aβ_1–42_, were counted for [^125^I] by using the Wallac Wizard^2^ 2470 automatic γ-counter (PerkinElmer). Unprocessed media was used to analyze [^14^C] via the Tri-Carb 2800 TR Liquid Scintillation Analyzer (PerkinElmer). The transcytosis quotient of intact [^125^I]-Aβ_1–42_ across the monolayer was calculated according to (luminal [^125^I]-Aβ_1–42_/input [^125^I]-Aβ_1–42_)/(luminal [^14^C]-inulin/input [^14^C]-inulin).

### Plaque load quantification

Mice were transcardially perfused with ice-cold PBS followed by 4% Roti^®^-Histofix (Roth). Brains were post-fixed overnight at 4 °C in 4% Roti^®^-Histofix, cryoprotected for 48 h at 4 °C in 30% (w/v) sucrose and stored at − 20 °C after shock freezing on dry ice. Tissues were processed as 30 µm-thick free-floating coronal sections (Leica CM3050 S). Washed sections were incubated for 3 min in 90% (v/v) formic acid for antigen retrieval and 3× for 10 min in TBS + 0.3% (v/v) Triton X-100 for permeabilization. After blocking in TBS + 0.3% (v/v) Triton X﻿-100 + 5% (w/v) BSA for 1 h sections were incubated with primary antibody overnight at 4 °C. Slices were washed before incubation with secondary antibody for 1 h, counterstained with 0.2 µg/ml DAPI (Sigma-Aldrich) and mounted on SUPERFROST^®^PLUS slides (ThermoFisher) using fluorescent mounting medium (Dako). Images were taken on a Zeiss 710 LSM confocal microscope at 10× magnification using 2 × 2 tile scan mode. Within one hemisphere 12 sections were analyzed; 6 representatives for prefrontal cortex (corresponding to bregma from   0.86 to + 0.62 mm) and 6 for the hippocampus (corresponding to bregma from − 1.82 to − 2.06 mm). Plaque load (aggregates ≥ 80 µm^2^) was quantified using the NIH ImageJ 1.52 software as described previously [50].

### Fear conditioning

24-week-old female 5xFAD mice *LRP1*^*BEfl/fl*^ or *LRP1*^*BE−/−*^ and wildtype age-matched female littermates treated as described earlier were assessed for contextual and cued fear response adapted to previous studies [39, 41], using the computerized TSE Multi Conditioning System (TSE GmbH). Movement was assessed by high-resolution infrared sensors (100 Hz) in three dimensions (14 mm distant) and documented by the FCS software. In the training phase, mice were placed in the conditioning chamber for 3 min, followed by a 30-s tone application (72 dB, 9 kHz). During the last 2 s, the tone was accompanied by shock delivery (0.7 mA). Contextual memory testing was performed 24 h-post conditioning, confronting the animals 3 min to the training context without tone application. Cued memory was assessed 2 h later, by pre-exposing the animals 3 min to a non-conditioned context, followed by a tone application for 60 s according to the training session. Freezing behavior (the absence of all but respiratory movements ≥ 2 s), motoric abilities, environmental behavior (% of explored area), and activity (≥ 30 mm/s)/hyperactivity (≥ 200 mm/s) levels were recorded automatically.

### Expression and purification of recombinant PCSK9

PCSK9 (UniProt: Q8NBP7, amino acid residues 31–692) was amplified from respective full-length cDNA (kindly provided from Jay Horton; UT Southwestern, USA) and cloned into pFastBac (Gibco) containing a meprin-α (UniProt: Q16819) signal peptide [51], followed by an N-terminal Strep-tag and a C-terminal Flag-tag. Virus production, protein expression and purification were carried out as described previously [51].

### Statistics

All experiments were repeated several times using multiple preparations of endothelial capillaries. The number of mice used for the in vivo studies was specified by the animal welfare authority. Data with the number of sample units and independent experimental runs indicated in each figure legend are depicted as means ± SEM. If necessary, data normalization was conducted as described in the respective figure legend. All statistical analyses were carried out using the GraphPad Prism 8.4 software. Unpaired two-tailed *t* tests and one-way ANOVA followed by Tukey correction for multiple comparisons (*α* = 0.05) were utilized as statistically appropriate. Statistical significance between groups was defined as *p* ˂ 0.05 (*) or *p* < 0.01 (**). Final images and schematics were created using CorelDRAW2018.

## Results

### PCSK9 decreases LRP1-mediated brain-to-blood [^125^I]-Aβ_1–42_-transport in vitro

To assess the regulatory influence of PCSK9 on LRP1-mediated transcytosis we compared Aβ clearance across endothelial monolayer in an established BBB model in vitro [14, 30]. Radiolabeled [^125^I]-Aβ_1–42_ in physiological concentrations (0.1 nmol/l) [31] and paracellular marker [^14^C]-inulin were applied to the abluminal compartment and transport across the endothelial monolayer was subsequently calculated by the transported amount of intact [^125^I]-Aβ_1–42_ into the luminal compartment in relation to diffused [^14^C]-inulin.

**Fig. 1:**
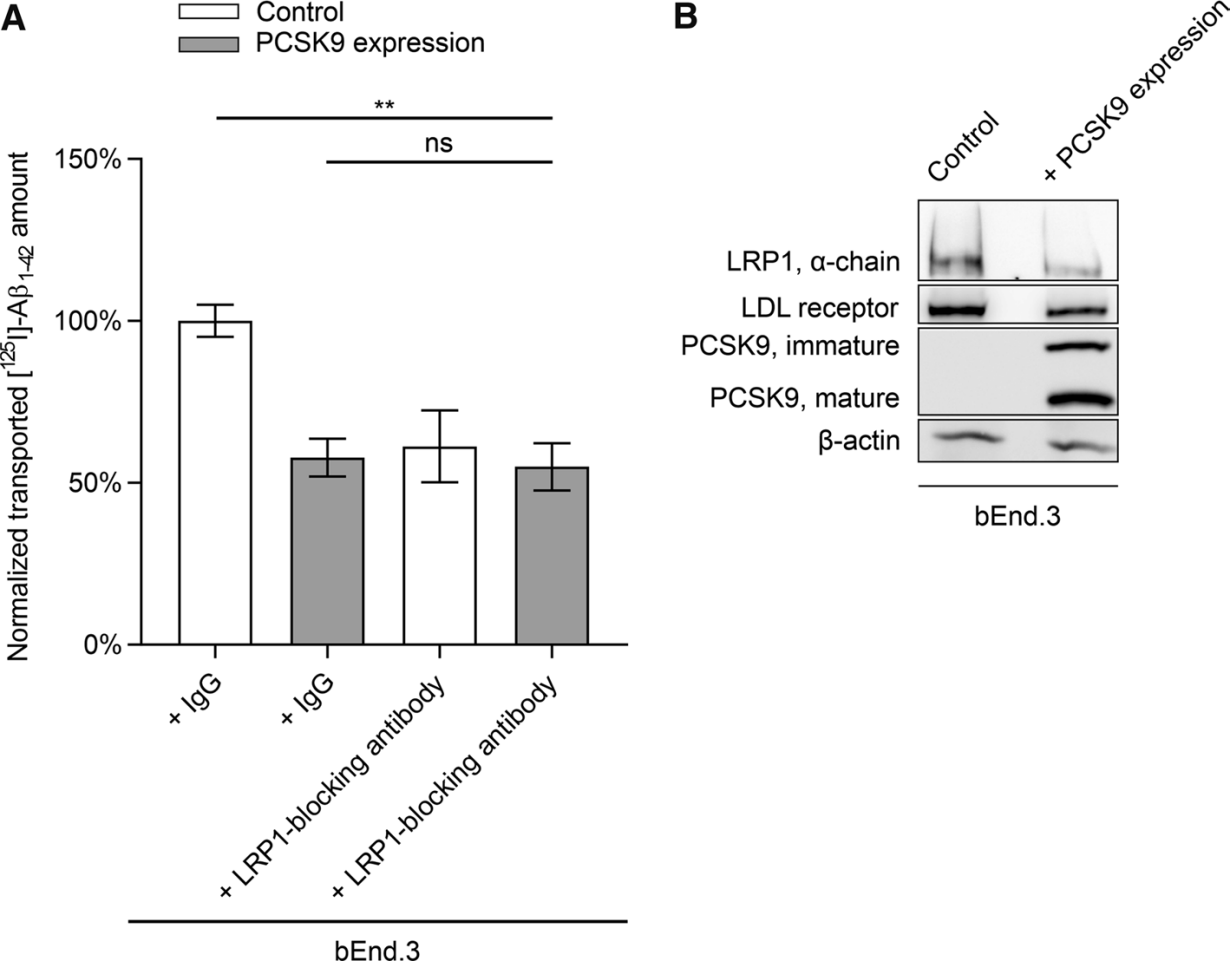
LRP1 inhibition do not further diminish [^125^I]-Aβ_1–42_ transcytosis across bEnd.3 cells stably expressing PCSK9. **A** Brain-to-blood clearance of 0.1 nmol/l [^125^I]-Aβ_1–42_ across bEnd.3 cell monolayer with or without PCSK9 expression was studied in the presence of [^14^C]-inulin as paracellular marker. Brain-to-blood transcytosis was analyzed by relating the luminal CPM for TCA-precipitable [^125^I]-radioactivity to the luminal DPM for [^14^C]-inulin. For LRP1 inhibition 15 µg/ml 11E2 antibody was applied to the abluminal compartment in comparison to 15 µg/ml non-specific mouse IgG. Transcytosis quotients were normalized to control mean (first column of graph section) and depicted as percentage of transport. Data represent mean ± SEM of *n* = 6–12 per group of at least two independent experiments. For statistical analyses one-way ANOVA followed by Tukey’s multiple comparison test was used (*p < 0.05; **p < 0.01). Data concerning inulin diffusion is provided in Supplementary Fig. 1A. **B** Cell lysate of naïve bEnd.3 cells and bEnd.3 cells stably transfected with PCSK9 were analyzed via immunoblot analyses for LDL receptor and LRP1 (α-chain) levels, as well as PCSK9 expression and representatively presented

bEnd.3 cells stably transfected with PCSK9 showed a ~ 42% reduction of transported [^125^I]-Aβ_1–42_ to the luminal compartment compared to naïve bEnd.3 cells (Fig. 1A). The reduction in [^125^I]-Aβ_1–42_ transport was similar to cells that were co-incubated with the established LRP1-blocking antibody 11E2 applied to the abluminal compartment (Fig. 1A). Co-incubation of 11E2 with bEnd.3 cells stably transfected with PCSK9 did not further reduce [^125^I]-Aβ_1–42_ transport (Fig. 1A), suggesting that Aβ transport inhibition by PCSK9 is mediated primarily by targeting cell surface LRP1. In line with these results, comparing bEnd.3 cells stably expressing PCSK9 with naïve cells revealed downregulated LRP1 as well as mature LDL receptor levels with PCSK9 expression (Fig. 1B).

**Fig. 2:**
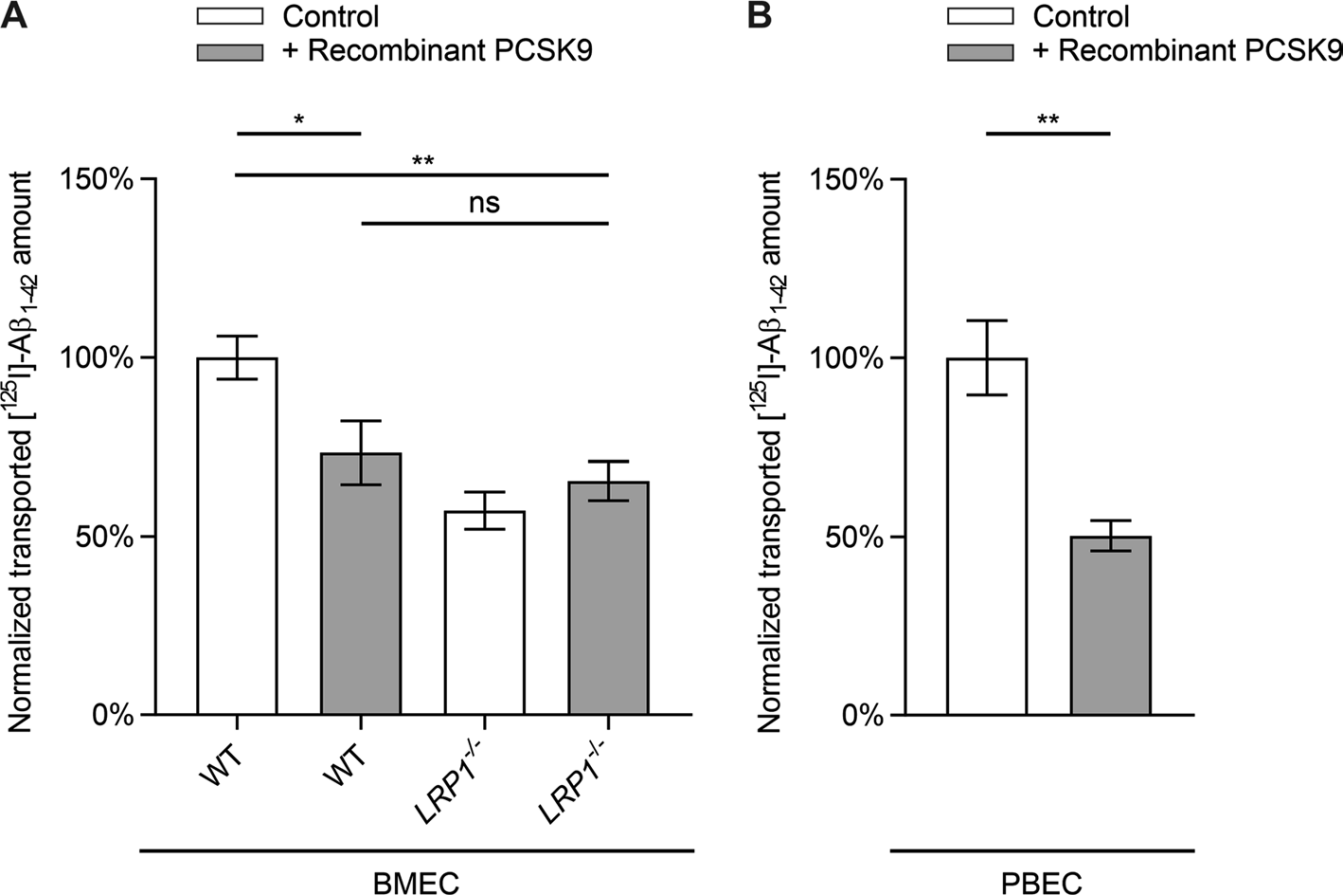
PCSK9 has no effect on [^125^I]-Aβ_1–42_ transport across BMECs *LRP1*^*−/−*^. Brain-to-blood clearance of 0.1 nmol/l [^125^I]-Aβ_1–42_ across **A** BMEC or **B** PBEC monolayer were studied in the presence of [^14^C]-inulin to normalize for passive diffusion. Brain-to-blood transcytosis was analyzed by relating the luminal CPM for TCA-precipitable [^125^I]-radioactivity to the luminal DPM for [^14^C]-inulin. Cells were treated 2 h before transport analyses with 5 µg/ml recombinant PCSK9 in luminal and abluminal compartment. Transcytosis quotients were normalized to control mean (first column of graph section) and depicted as percentage of transport. Data represent mean ± SEM of **A** *n* = 8–13 or **B** *n* = 9–10 per group of at least two independent experiments. For statistical analyses **A** one-way ANOVA followed by Tukey’s multiple comparison test or **B** unpaired two-tailed *t*-test were used (*p < 0.05; **p < 0.01). Data concerning inulin diffusion is provided in Supplementary Fig. 1B–D

In order to confirm that PCSK9 is mainly targeting LRP1 in our transport assay, we used BMECs expressing or lacking LRP1 (*LRP1*^*−/−*^) [14]. Treating wildtype BMECs with recombinant PCSK9 results in a ~ 27% reduction in brain-to-blood transport of [^125^I]-Aβ_1–42_ across the endothelial monolayer compared to control-treated BMECs (Fig. 2A). The PCSK9-mediated reduction in [^125^I]-Aβ_1–42_ transport was similar to transport levels detected in control-treated BMECs *LRP1*^*−/−*^ (Fig. 2A). Consistent with our previous observations, extracellular treatment with PCSK9 did not further diminish [^125^I]-Aβ_1–42_ transcytosis across BMECs *LRP1*^*−/−*^ (Fig. 2A), confirming the hypothesis that extracellular PCSK9 downregulates Aβ transcytosis in a cell surface LRP1-dependent fashion.

Treating PBECs in a similar manner, PCSK9 reduces brain-to-blood transport of [^125^I]-Aβ_1–42_ by ~ 50% (Fig. 2B), indicating a PCSK9-induced regulatory mechanism of Aβ clearance across primary brain endothelial cells of different species.

Investigating the distribution of the paracellular marker [^14^C]-inulin revealed in all our transport studies that the presence of PCSK9 is not affecting BBB integrity, substantiating its regulatory effect on receptor-mediated endocytosis in these experimental setups (Supplementary Fig. 1).

### PCSK9 inhibition decreases cerebral Aβ burden and preserves cognitive capacities in vivo

To evaluate the impact of PCSK9 on Aβ clearance and the potential participation of brain endothelial LRP1 in this mechanism in vivo we repetitively injected Alirocumab or Evolocumab—commercially available PCSK9 inhibitors—i.p. into 14-week-old hemizygous female 5xFAD mice harboring a tamoxifen-inducible brain endothelial LRP1 knock-out for 10 weeks.

**Fig. 3:**
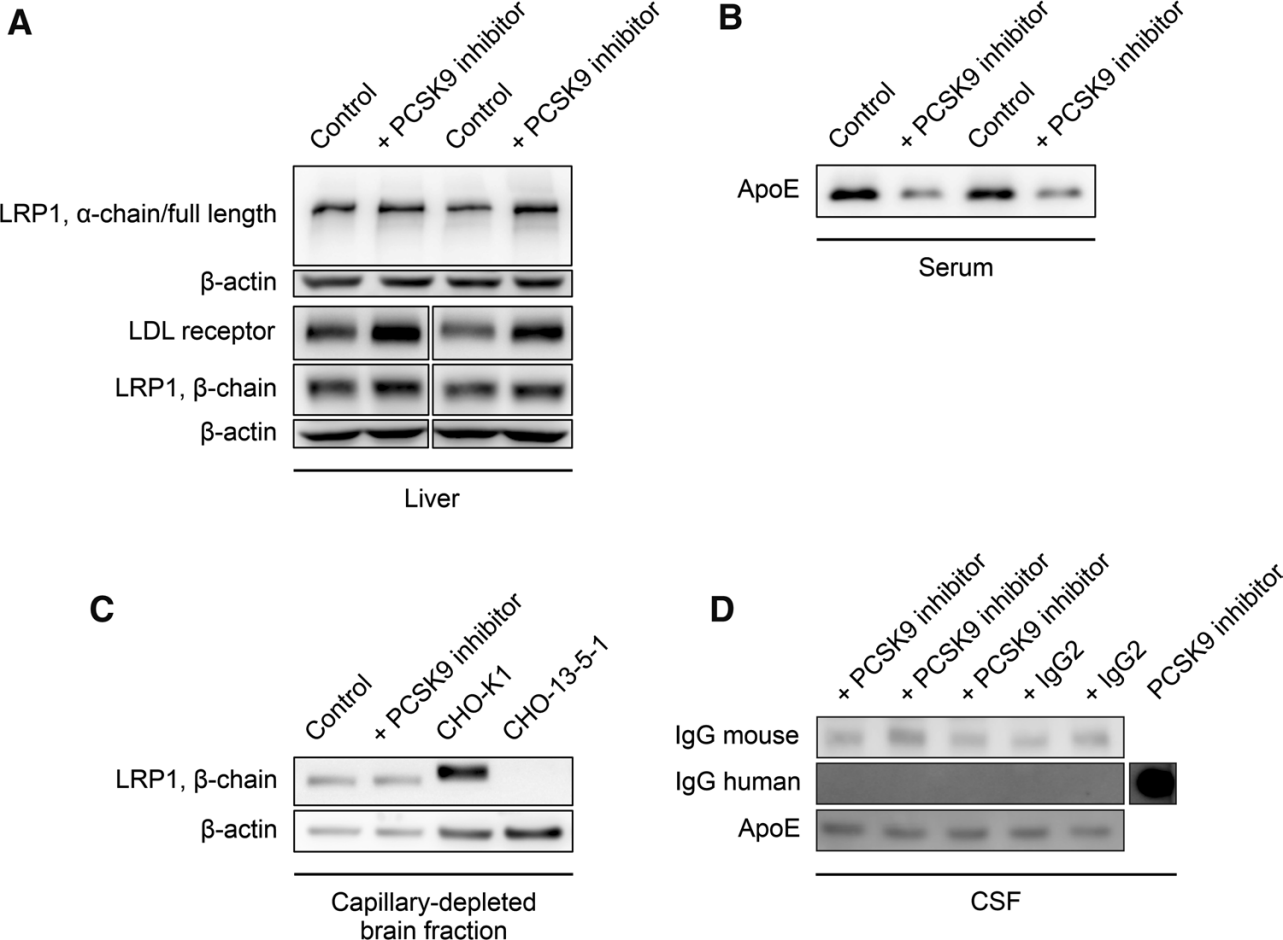
Systemic PCSK9 inhibition increases peripheral LRP1 level. **A** Liver lysates, **B** serum, as well as **C** capillary-depleted brain fractions and **D** CSF samples of 6-month-old 5xFAD mice, treated repetitively with **A–C** 1 µg/g Alirocumab or **D** Evolocumab in comparison to **A–C** 0.9% NaCl or **D** non-specific human control IgG2 for 10 weeks were evaluated via immunoblot analyses for LRP1 (full length, α-chain, β-chain), LDL receptor, human as well as mouse IgG, and ApoE, and representatively presented. **A** Lanes were run on the same gel but were noncontinuous. **C** LRP1 signals were verified by loading cell lysate of CHO-K1 (LRP1^+^) and the chemically modified CHO-K1 cell line CHO-13–5-1 (LRP1^−^). **D** Evolocumab antibody was loaded to represent and verify human IgG signal

To address target recognition of PCSK9, we monitored peripheral LRP1 and LDL receptor levels by analyzing liver lysates and detected increased LRP1 and LDL receptor concentrations (Fig. 3A). Furthermore, systemic PCSK9 inhibition significantly reduces serum LDL/VLDL- (Supplemental Fig. 2A), but not HDL-cholesterol levels (Supplemental Fig. 2B) [52, 53], and substantially decreases the amount of free circulating PCSK9 (Supplemental Fig. 2C) compared to control-treated mice. In line with higher peripheral ApoE receptor concentrations in the liver (LRP1 and LDL receptor), serum levels of ApoE were also strongly reduced in PCSK9 inhibitor-treated 5xFAD animals (Fig. 3B).

In contrast to peripheral LRP1 and circulating ApoE, analysis of capillary-depleted brain fractions revealed that cerebral LRP1 seems to be unaffected by systemic PCSK9 inhibition (Fig. 3C) as well as CSF ApoE levels (Fig. 3D). Furthermore, we were not able to detect traces of therapeutic antibody or non-specific control IgG2 within the CSF and endogenous IgG levels were similar among the treatment groups (Fig. 3D), indicating similar blood–brain barrier integrities [54, 55].

**Fig. 4:**
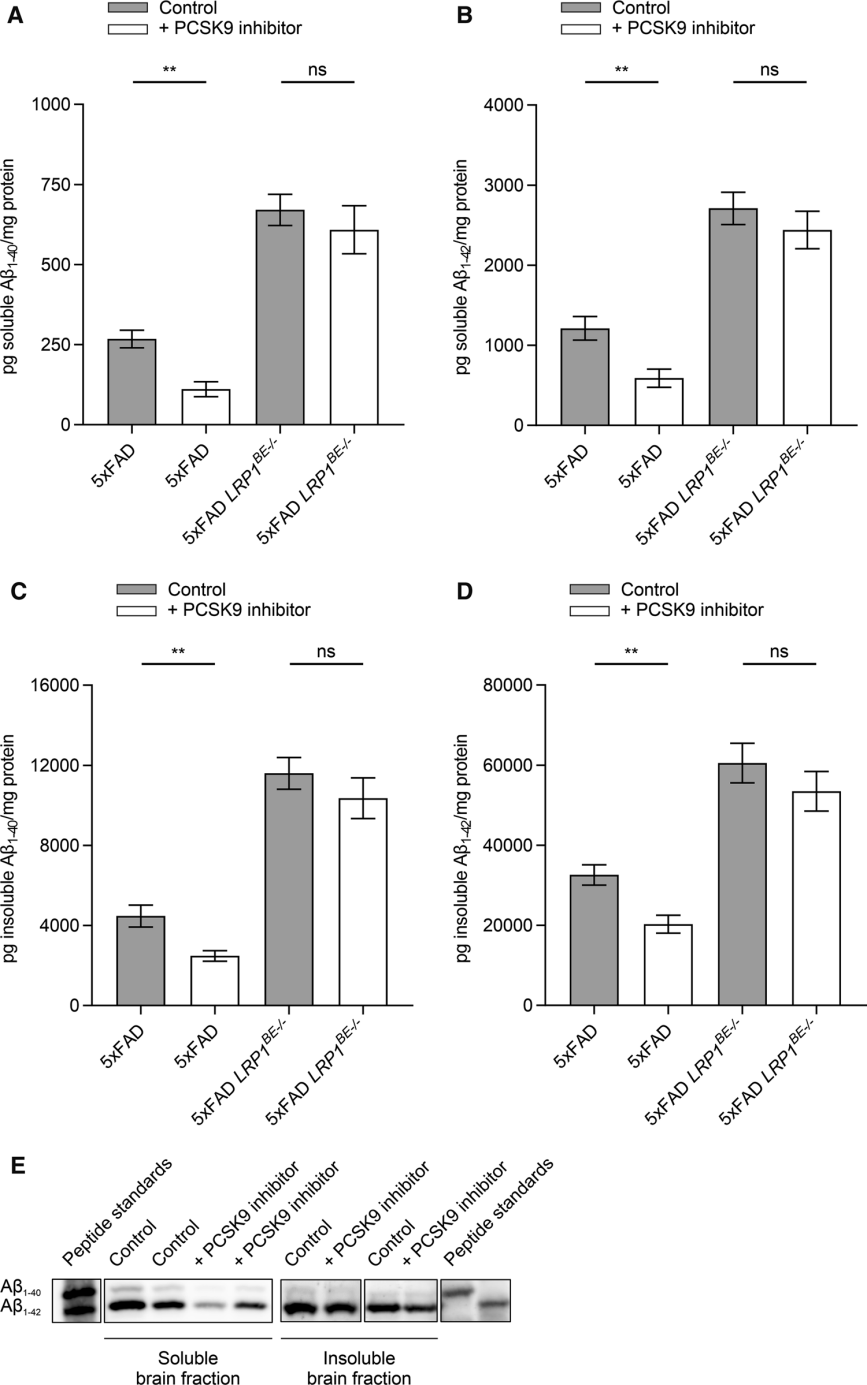
Peripheral PCSK9 inhibition reduces overall Aβ burden. **A** + **B** Soluble and **C** + **D** insoluble brain **A** + **C** Aβ_1–40_ or **B** + **D** Aβ_1–42_ level of 6-month-old 5xFAD or 5xFAD *LRP1*^*BE−/−*^ mice, treated, repetitively, with 1 µg/g Alirocumab or 0.9% NaCl for 10 weeks were assessed via ELISA (**E**) and representatively displayed by high-resolution urea SDS-PAGE. Lanes were run on the same gel but were noncontinuous. Aβ_1–40_ and Aβ_1–42_ signals were verified by loading peptide standards. Data represent mean ± SEM of *n* = 4–14 mice per group. For statistical analyses one-way ANOVA followed by Tukey’s multiple comparison test was used (*p < 0.05; **p < 0.01)

**Fig. 5:**
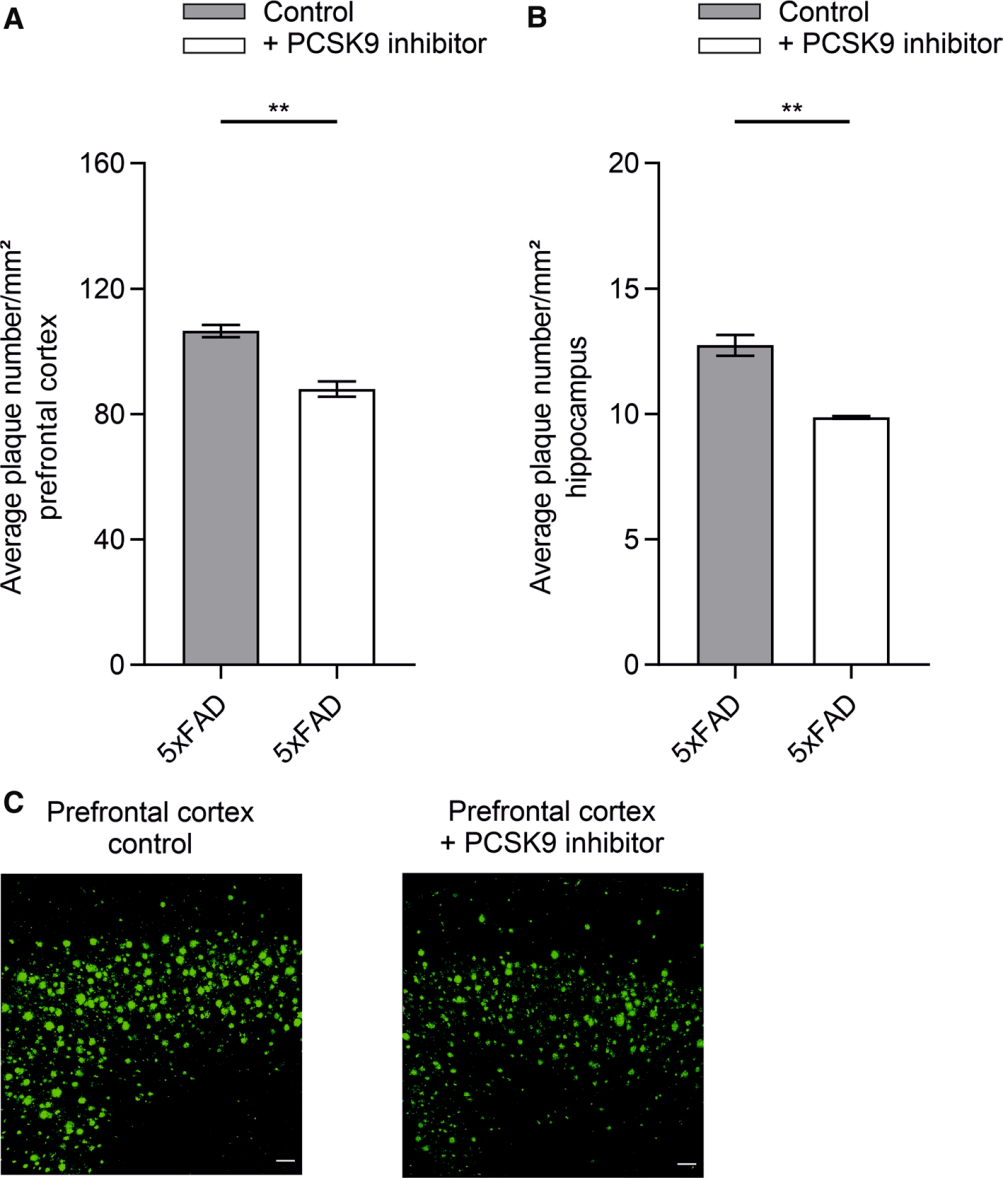
Aβ plaque load of prefrontal cortex and hippocampus is reduced due to systemic PCSK9 inhibition. **A** Prefrontal cortex and **B** hippocampus of 6-month-old 5xFAD mice treated with 1 µg/g alirocumab or 0.9% NaCl for ten weeks were quantified for amyloid plaques. Aβ-specific fluorescence of plaques (≥ 80 µm^2^) of six representative sections per hemisphere were assessed—unaware of the specific treatment—via confocal microscopy at ×10 magnification using 2 × 2 tile scan mode and averaged per mouse. **C** The plaque burden of prefrontal cortex was representatively displayed. Scale bars represent 100 μm. Data represents mean ± SEM of **A** *n* = 8 or **B** *n* = 3–4 mice per group. For statistical analyses unpaired two-tailed *t* test was used (*p < 0.05; **p < 0.01)

PCSK9 inhibition by Alirocumab resulted in a significant reduction in cerebral Aβ_1–40_ and Aβ_1–42_ levels, which were determined by ELISA and representatively visualized via high-resolution urea SDS-PAGE (Fig. 4A–E). Peripheral inhibition of PCSK9 reduced the amount of soluble Aβ_1–40_ and Aβ_1–42_ by ~ 58% (Fig. 4A) and ~ 51% (Fig. 4B), and insoluble Aβ_1–40_ and Aβ_1–42_ by ~ 44% (Fig. 4C) and ~ 38% (Fig. 4D), respectively, compared to brain fractions of control-treated mice. Plaque load quantifications of prefrontal cortex and hippocampus sections of 5xFAD mice treated with Alirocumab and control-treated animals revealed that even highly aggregated Aβ was significantly reduced by ~ 17% (Fig. 5A + C) and ~ 23% (Fig. 5B), respectively. In contrast, 5xFAD *LRP1*^*BE−/−*^ mice display no significant difference of soluble and insoluble Aβ_1–40_ and Aβ_1–42_ levels when treated with PCSK9 inhibitors compared to control-treated 5xFAD *LRP1*^*BE−/−*^ mice (Fig. 4A–D), substantiating the hypothesis that PCSK9-regulated Aβ clearance depends on brain endothelial LRP1.

**Fig. 6:**
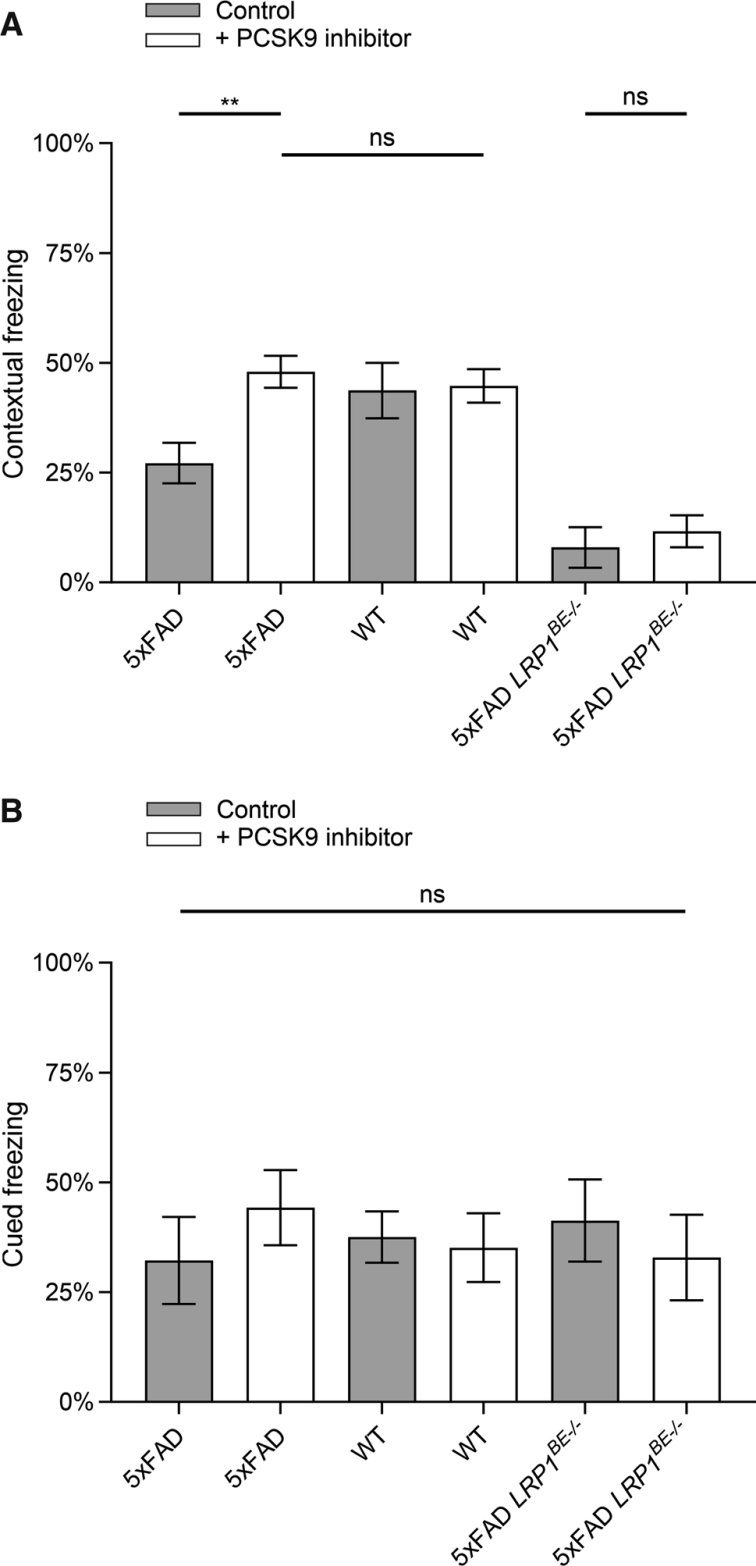
Peripheral PCSK9 inhibition improves contextual memory formation of 5xFAD transgenic mice in a Fear Conditioning paradigm. 6-month-old 5xFAD mice treated with 1 µg/g Alirocumab or 0.9% NaCl for 10 weeks were analyzed for **A** hippocampus-dependent contextual fear response and **B** hippocampus-independent cued fear response in comparison to wildtype and *LRP1*^*BE−/−*^ littermates. Fear was assessed by freezing behavior (the absence of all but respiratory movements), when confronted again to the **A** context or **B** tone of the training session and displayed as percentage of the measurement period (**A** 31–180 s, **B** 1–60 s). Higher freezing behavior percentage indicates stronger memory formation. Data represent mean ± SEM of *n* = 8–13 mice per group. For statistical analyses one-way ANOVA followed by Tukey’s multiple comparison test was used (*p < 0.05; **p < 0.01)

5xFAD mice show hippocampus-dependent memory deficits at 4–6 months of age compared to wildtype littermates which can be detected by a contextual fear conditioning paradigm [39]. We used the established experimental setup to assess whether the reduced cerebral Aβ burden in 6-month-old 5xFAD mice treated with Alirocumab correlates with improved memory formation. The successful association between context and cue with the unconditioned aversive stimulus, evokes a conditioned fear response displayed by freezing behavior, when confronted again with the conditioned stimulus. After a single pairing of conditioned and unconditioned stimulus, 5xFAD animals treated with PCSK9-inhibitors display a significant increased contextual fear response compared to control-treated 5xFAD mice 24 h post-training (Fig. 6A). In comparison to control-treated wildtype littermates no significant difference in contextual freezing was detectable (Fig. 6A), indicating that peripheral PCSK9 inhibition preserves hippocampus-dependent learning capacities of 5xFAD mice. Since we did not detect any significant difference between the differently treated wildtype animal groups (Fig. 6A), we concluded that the observed positive influence on memory formation is restricted to Aβ-induced neurodegeneration in this AD mouse model. In contrast, 5xFAD *LRP1*^*BE−/−*^ mice do not significantly benefit from PCSK9 inhibition, revealing a reduced hippocampus-dependent learning capacity irrespective of treatment type (Fig. 6A). To assess whether systemic PCSK9 inhibition is targeting specifically Aβ-induced hippocampus deficits we assessed 2 h after contextual fear the hippocampus-independent cued fear response of the same animals [41]. In accordance with previous experiments [41] no significant differences between 5xFAD, 5xFAD *LRP1*^*BE−/−*^, and wildtype littermates were observed, irrespective of PCSK9-inhibition or control treatment (Fig. 6B). Considering that we did not detect any significant difference due to PCSK9 inhibition concerning motoric abilities, activity or hyperactivity levels, environmental behavior, or basal freezing during the conditioning phase (Supplementary Fig. 3, Supplementary Fig. 4), our data suggests that peripheral PCSK9 inhibition prevents Aβ-induced hippocampus deficits and that brain endothelial LRP1 is an essential component of this mechanism.

## Discussion

The increase in soluble Aβ oligomers in the brain is hypothesized to drive neurodegenerative decline in AD [3]. In non-pathological conditions the clearance of cerebral Aβ exceeds its production [9]. However, AD patients show decreased Aβ clearance accompanied by an unaffected production rate [10]. LRP1 is a key mediator of cerebral Aβ clearance by its rapid Aβ transport across the BBB into the periphery [13, 14]. According to the peripheral sink hypothesis efficient LRP1-mediated clearance of Aβ in the periphery might further promote the reduction of cerebral Aβ due to a reduced transition of circulating Aβ peptides into the brain [56]. Consistently, LRP1 expression is decreasing with age in brain microvasculature [57, 58] and liver [19], while ageing increases the incidence of the progressive neurodegenerative disease [59]. Levels of LDL receptor family members, including LRP1, are negatively regulated by the secreted serine protease PCSK9 [20–22], but the consequences of LRP1 regulation by circulating PCSK9 are less well studied.

To investigate the influence of PCSK9 on LRP1-mediated transport we used our established in vitro BBB model [14, 30] with either bEnd.3 cells stably expressing PCSK9 (Fig. 1), PCSK9-treated BMECs (Fig. 2A), or PCSK9-treated PBECs (Fig. 2B). Analyzing the transported amount of radiolabeled [^125^I]-Aβ_1–42_ across the endothelial monolayer revealed a dramatic reduction in brain-to-blood [^125^I]-Aβ_1–42_ clearance due to PCSK9 in a LRP1-dependent mechanism. Taking into consideration that we do not detect any impact of PCSK9 on BBB integrity during these transport studies (Supplementary Fig. 1), our data indicate that extracellular PCSK9 targets LRP1 at the surface of endothelial cells and reduces LRP1-mediated Aβ brain clearance.

To evaluate whether therapeutic targeting of peripheral PCSK9 would increase Aβ brain clearance in vivo, we i.p. injected FDA-approved monoclonal anti-PCSK9 antibodies repetitively into the AD mouse model 5xFAD to inhibit endogenous PCSK9 activity. Consequently, we observed that the injection of low-concentrated Alirocumab for 10 weeks in 5xFAD mice significantly reduces Aβ pathology compared to control-treated 5xFAD mice (Figs. 4, 5) without inducing general behavioral changes (Fig. 6B, Supplementary Fig. 3, Supplementary Fig. 4). To analyze the potential role of endothelial LRP1 at the BBB on PCSK9-regulated Aβ clearance we used our previously established brain endothelial-specific LRP1 knock-out model [14]. Treating 5xFAD *LRP1*^*BE−/−*^ mice analogously with PCSK9 inhibitors did not result in increased cerebral Aβ clearance compared to control-treated mice, which might indicate the important role of brain endothelial LRP1 in this regulatory mechanism and its accessibility for peripheral treatment options (Fig. 4A–D).

AD is characterized by a progressive loss of memory functions [60]. In accordance with detectable reduced Aβ pathologies in prefrontal cortex (Fig. 5A + C) and hippocampus (Fig. 5B)—brain structures, which are essentially participating in memory formation and retrieval [61, 62] -, targeting peripheral PCSK9 in 5xFAD mice preserves hippocampus-dependent learning capacities [39] to a level of control-treated wildtype littermates (Fig. 6A). Since wildtype and 5xFAD *LRP1*^*BE−/−*^ mice did not display a significant intensified contextual freezing behavior due to PCSK9 inhibition compared to control (Fig. 6A), we concluded that the increase in hippocampus-dependent memory formation is specifically attributed to the inhibited PCSK9 downregulation of LRP1-mediated Aβ clearance.

Because it has been shown that blood-circulating PCSK9 is not capable of crossing an intact BBB [63], we postulate that peripheral PCSK9 inhibition modulates cerebral Aβ burden via brain endothelial and peripheral LRP1 levels. Even though our data display a substantial impact of PCSK9 on endothelial LRP1 activity, we cannot attribute the significant reduction of cerebral Aβ and cognitive improvement in vivo solely to brain endothelial LRP1. The dramatic impact of *LRP1*^*BE−/−*^ on Aβ pathology (Fig. 4A–D) and hippocampus-dependent learning behavior (Fig. 6A), might mask further potential beneficial effects of peripheral PCSK9 inhibition. Since we were detecting increased LRP1 levels in the liver as well (Fig. 3A), it is conceivable that strengthened peripheral clearance mechanisms, for instance via hepatic LRP1, contribute to the improved Aβ clearance as well, which is in line with the postulated peripheral sink hypothesis [56].

In contrast to ongoing therapeutic efforts to reduce the cerebral Aβ burden via anti-Aβ antibodies, which have to cross the highly-selective BBB, our study suggests that overcoming the BBB might not be ultimately necessary if the peripheral administration of an anti-PCSK9 antibody might be already sufficient to enhance Aβ brain clearance and preserve memory function (Fig. 3D﻿). Considering that the application of these antibodies is an already established therapy used to treat severe hypercholesterolemia with little adverse effects [27], peripheral PCSK9 inhibition could be a valuable tool to target cerebral Aβ accumulation in AD patients. In general, PCSK9 might be a key player in promoting neurodegeneration, since there is evidence that elevated PCSK9 levels in the CSF c﻿orrelate with AD patients [64] and neurodegenerative disorders in general [65]. It has to be mentioned that an anti-PCSK9 antibody study observed no significant between-group difference in cognitive function over a median of 19 months [66]. However, these tests were designed to monitor for potential short-term side effects due to PCSK9 inhibition and not for the investigation of cognitive decline progression in a long-term AD therapy. In fact, it is suggested that anti-Aβ therapies should start decades before the onset of clinical symptoms to have significant influences on cognitive decline [67–69]. Since the first anti-PCSK9 antibody Evolocumab was approved in 2015, prospective long-time studies investigating the effect of PCSK9 inhibition on cerebral Aβ burden and cognitive functions will show if these antibodies effectively enhance Aβ brain clearance in humans and could be a potential treatment for AD.

Nevertheless, this study strongly supports the idea that peripheral PCSK9 inhibition could be a novel and easily implementable tool to increase cerebral Aβ clearance, thus targeting Aβ brain accumulation in AD with already available FDA-approved PCSK9 inhibitors.

## Supplementary Information

Below is the link to the electronic supplementary material.

Supplementary file1 (PDF 797 KB)

Supplementary file2 (DOCX 244 KB)

Supplementary file3 (DOCX 369 KB)

Supplementary file4 (PDF 609 KB)

## Data Availability

The datasets generated and/or analyzed during the current study are available from the corresponding author on reasonable request.

## Abbreviations

Aβ: Amyloid-β
AD: Alzheimer’s disease
ApoE: Apolipoprotein E
BBB: Blood–brain barrier
BMECs: Brain mouse endothelial cells
CSF: Cerebrospinal fluid
FAD: Familial Alzheimer’s disease
LDL: Low-density lipoprotein
LRP1: Low-density lipoprotein receptor-related protein 1
PBECs: Porcine brain endothelial cells
PCSK9: Proprotein convertase subtilisin/kexin type 9
