# Supplementary material for: PCSK9 acts as a key regulator of Aβ clearance across the blood–brain barrier
Source: Cell Mol Life Sci. 2022 Mar 27;79(4):212. doi: 10.1007/s00018-022-04237-x (PMC8960591; doi:10.1007/s00018-022-04237-x)
Supplement: Supplementary file 1 — Supplementary file1 (PDF 797 KB) [file 18_2022_4237_MOESM1_ESM.pdf]

TITLE: PCSK9 acts as a key regulator of Aβ clearance across the blood-brain barrier

Cellular and Molecular Life Sciences

AUTHORS: Alexander D. Mazura[1] (0000-0002-2899-6183), Anke Ohler[1], Steffen E. Storck[1] (0000-0002-6965-2264), Magdalena Kurtyka[1], Franka Scharfenberg[2], Sascha Weggen[3], Christoph Becker-Pauly[2], Claus U. Pietrzik[1]

AFFILIATIONS

[1]Institute of Pathobiochemistry, University Medical Center of the Johannes Gutenberg-University Mainz; Mainz, 55128, Germany.

[2]Institute of Biochemistry, Christian Albrecht University of Kiel; Kiel, 24098, Germany.

[3]Department of Neuropathology, Heinrich Heine University Düsseldorf; Düsseldorf, 40225, Germany.

Corresponding author: Claus U. Pietrzik, Institute of Pathobiochemistry, University Medical Center of the Johannes Gutenberg-University Mainz; Duesbergweg 6., 55128 Mainz, Germany; Phone: +49 6131 39 25390; Email: pietrzik@uni-mainz.de

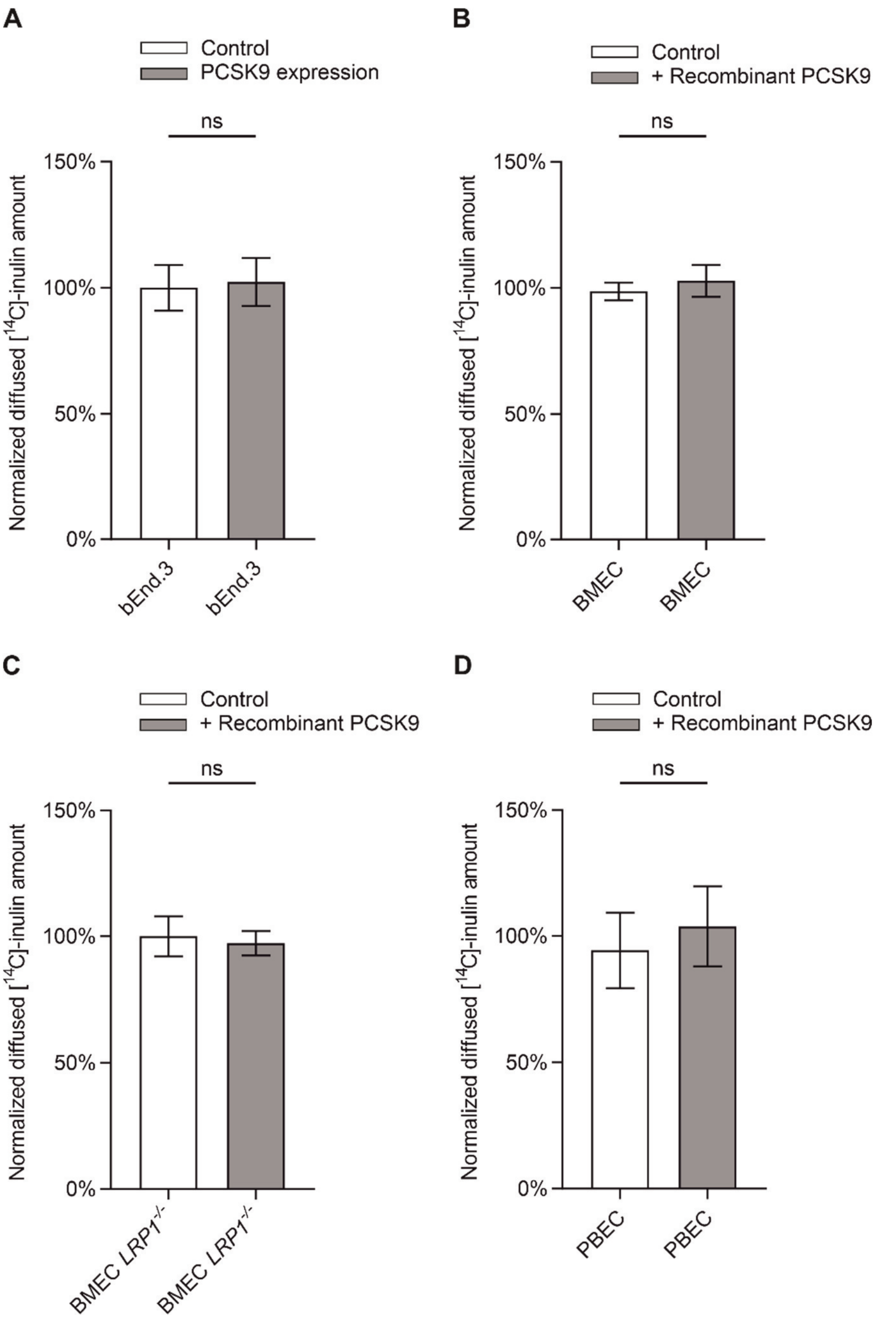

Supplementary Fig. 1 No substantial differences in BBB integrity *in vitro*. Luminal [^{14}C]-radioactivity after conducting the *in vitro* transport studies displayed in (**A**) Fig. 1A and (**B - D**) 2A + B were normalized to control mean (first column of graph section) and depicted as percentage of inulin diffusion. Data represents mean ± SEM of (A) n = 6 - 12, (B + C) n = 8 - 13, or (D) n = 9 - 10 per group of at least two independent experiments. For statistical analyses unpaired two-tailed t-test was used (*p <0.05)
