# Supplementary material for: PCSK9 acts as a key regulator of Aβ clearance across the blood–brain barrier
Source: Cell Mol Life Sci. 2022 Mar 27;79(4):212. doi: 10.1007/s00018-022-04237-x (PMC8960591; doi:10.1007/s00018-022-04237-x)
Supplement: Supplementary file 2 — Supplementary file2 (DOCX 244 KB) [file 18_2022_4237_MOESM2_ESM.docx]

**TITLE: PCSK9 acts as a key regulator of Aβ clearance across the blood‑brain barrier**

*Cellular and Molecular Life Sciences*

**AUTHORS:** Alexander D. Mazura^1^ **(0000-0002-2899-6183)**, Anke Ohler^1^, Steffen E. Storck^1^ (0000-0002-6965-2264), Magdalena Kurtyka^1^, Franka Scharfenberg^2^, Sascha Weggen^3^, Christoph Becker‑Pauly^2^, Claus U. Pietrzik^1^

**AFFILIATIONS**

^1^Institute of Pathobiochemistry, University Medical Center of the Johannes Gutenberg-University Mainz; Mainz, 55128, Germany.

^2^Institute of Biochemistry, Christian Albrecht University of Kiel; Kiel, 24098, Germany.

^3^Department of Neuropathology, Heinrich Heine University Düsseldorf; Düsseldorf, 40225, Germany.

Corresponding author: Claus U. Pietrzik, Institute of Pathobiochemistry, University Medical Center of the Johannes Gutenberg‑University Mainz; Duesbergweg 6., 55128 Mainz, Germany; Phone: +49 6131 39 25390; Email: [pietrzik@uni-mainz.de](mailto:pietrzik@uni-mainz.de)

**
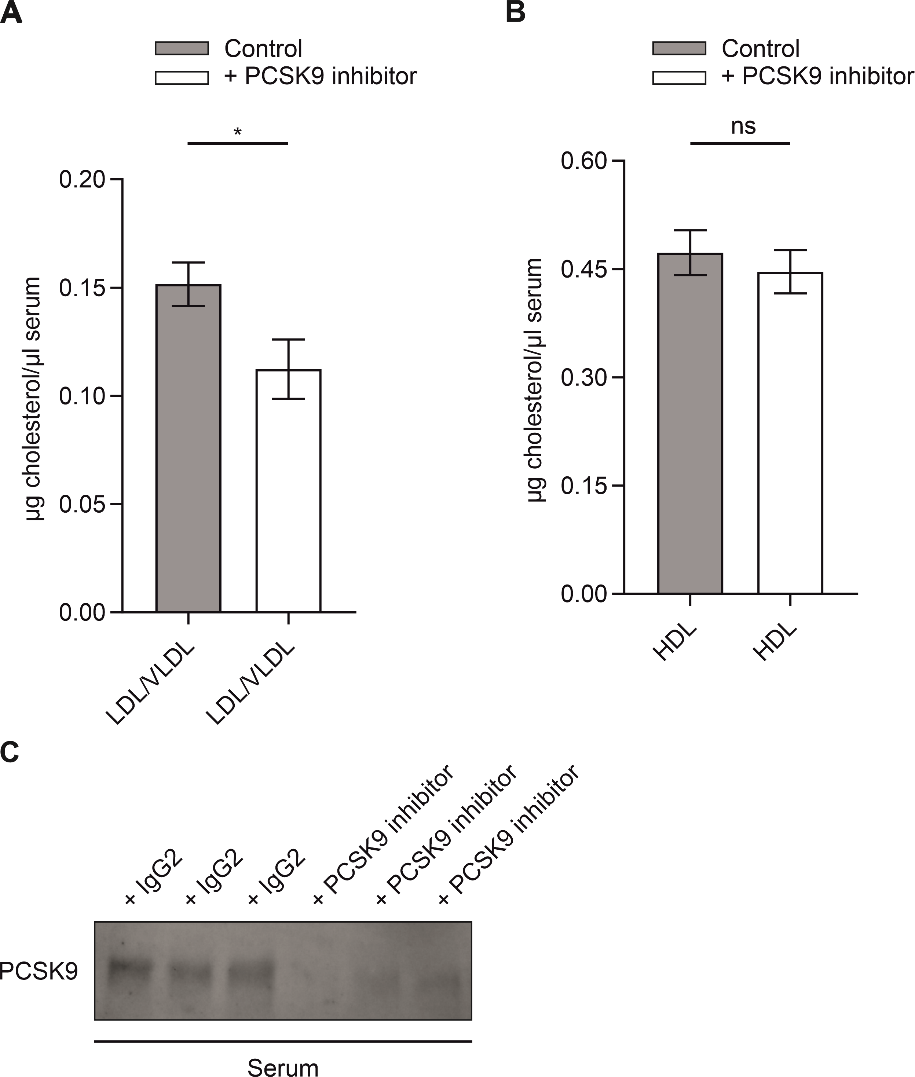
**

**Supplementary Fig. 2 Systemic PCSK9 inhibition reduces serum LDL/VLDL and free PCSK9 levels.** (**A**) Serum LDL/VLDL‑ and (**B**) HDL‑cholesterol levels of 6‑months-old 5xFAD mice, treated repetitively with 1 µg/g Evolocumab or non‑specific human IgG2 control antibody for ten weeks were assessed via ELISA. Data represents mean ± SEM of *n* = 8 ‑ 9 mice per group. For statistical analyses unpaired two‑tailed *t*‑test was used (**p* <0.05). (**C**) Additionally, serum samples were precleared by using raw Protein A Agarose and incubated with Protein A Agarose crosslinked to Evolocumab. Levels of immunoprecipitated PCSK9 were evaluated via immunoblot analyses and representatively presented
