# Supplementary material for: PCSK9 acts as a key regulator of Aβ clearance across the blood–brain barrier
Source: Cell Mol Life Sci. 2022 Mar 27;79(4):212. doi: 10.1007/s00018-022-04237-x (PMC8960591; doi:10.1007/s00018-022-04237-x)
Supplement: Supplementary file 3 — Supplementary file3 (DOCX 369 KB) [file 18_2022_4237_MOESM3_ESM.docx]

**TITLE: PCSK9 acts as a key regulator of Aβ clearance across the blood‑brain barrier**

*Cellular and Molecular Life Sciences*

**AUTHORS:** Alexander D. Mazura^1^ **(0000-0002-2899-6183)**, Anke Ohler^1^, Steffen E. Storck^1^ (0000-0002-6965-2264), Magdalena Kurtyka^1^, Franka Scharfenberg^2^, Sascha Weggen^3^, Christoph Becker‑Pauly^2^, Claus U. Pietrzik^1^

**AFFILIATIONS**

^1^Institute of Pathobiochemistry, University Medical Center of the Johannes Gutenberg-University Mainz; Mainz, 55128, Germany.

^2^Institute of Biochemistry, Christian Albrecht University of Kiel; Kiel, 24098, Germany.

^3^Department of Neuropathology, Heinrich Heine University Düsseldorf; Düsseldorf, 40225, Germany.

Corresponding author: Claus U. Pietrzik, Institute of Pathobiochemistry, University Medical Center of the Johannes Gutenberg‑University Mainz; Duesbergweg 6., 55128 Mainz, Germany; Phone: +49 6131 39 25390; Email: [pietrzik@uni-mainz.de](mailto:pietrzik@uni-mainz.de)

**
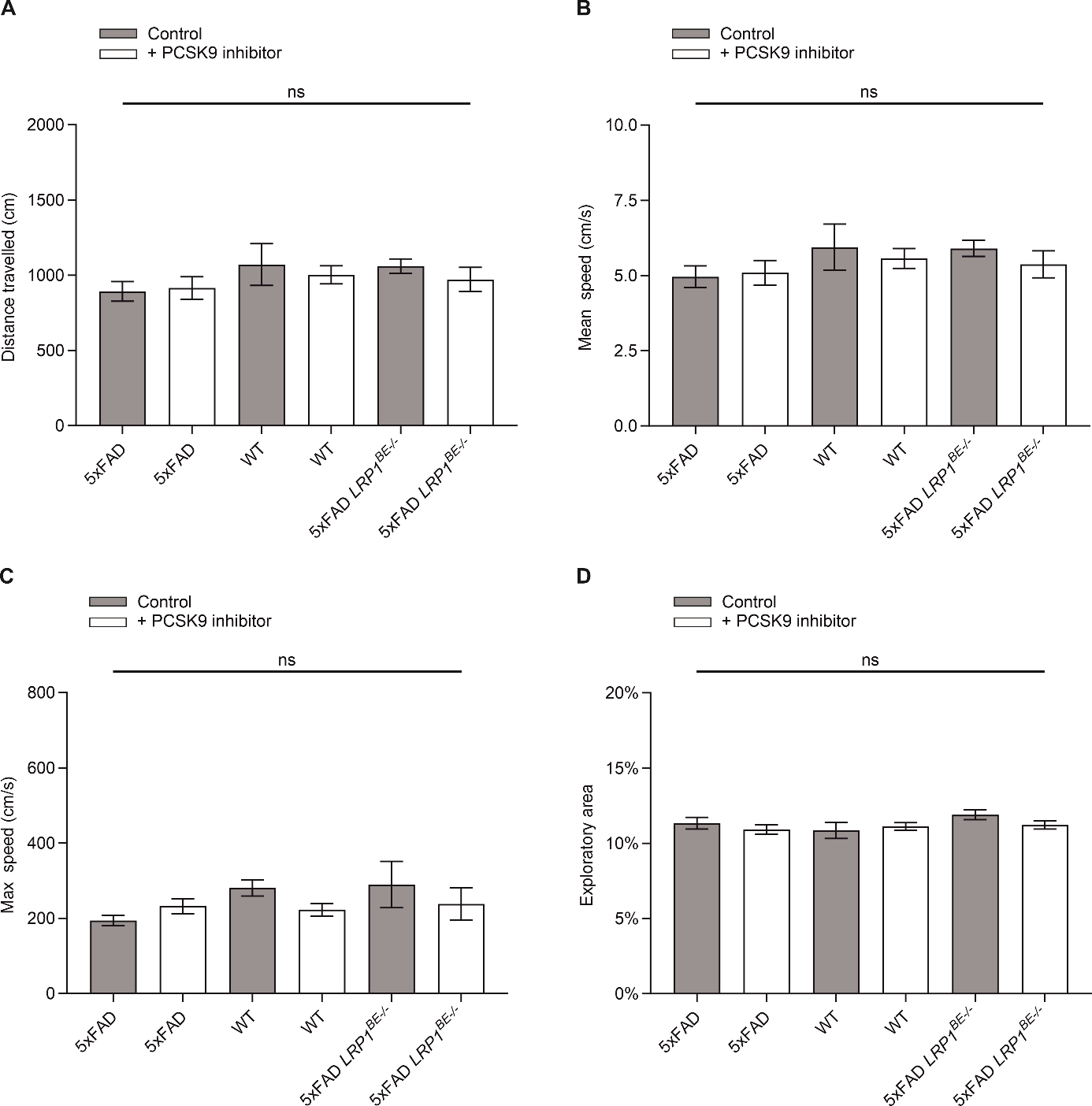
**

**Supplementary Fig. 3 No differences in motoric abilities or environmental behavior during the conditioning phase.** 6-months old 5xFAD mice treated with 1 µg/g Alirocumab or 0.9% NaCl for ten weeks used for Fear Conditioning experiments were analyzed for (**A - C**) motoric abilities and (**D**) environmental behavior during the training phase. Data represents mean ± SEM of *n* = 8 ‑ 13 mice per group. For statistical analyses one-way ANOVA followed by Tukey‘s multiple comparison test was used (**p* <0.05)
